# Physical Map of FISH 5S rDNA and (AG_3_T_3_)_3_ Signals Displays *Chimonanthus campanulatus* R.H. Chang & C.S. Ding Chromosomes, Reproduces its Metaphase Dynamics and Distinguishes Its Chromosomes

**DOI:** 10.3390/genes10110904

**Published:** 2019-11-07

**Authors:** Xiaomei Luo, Jingyuan Chen

**Affiliations:** College of Forestry, Sichuan Agricultural University, Huimin Road 211, Wenjiang District, Chengdu 611130, China; 18482004340@163.com

**Keywords:** mitotic metaphase, satellite chromosome, physical map

## Abstract

*Chimonanthus campanulatus* R.H. Chang & C.S. Ding is a good horticultural tree because of its beautiful yellow flowers and evergreen leaves. In this study, fluorescence in situ hybridization (FISH) was used to analyse mitotic metaphase chromosomes of *Ch. campanulatus* with 5S rDNA and (AG_3_T_3_)_3_ oligonucleotides. Twenty-two small chromosomes were observed. Weak 5S rDNA signals were observed only in proximal regions of two chromosomes, which were adjacent to the (AG_3_T_3_)_3_ proximal signals. Weak (AG_3_T_3_)_3_ signals were observed on both chromosome ends, which enabled accurate chromosome counts. A pair of satellite bodies was observed. (AG_3_T_3_)_3_ signals displayed quite high diversity, changing in intensity from weak to very strong as follows: far away from the chromosome ends (satellites), ends, subtelomeric regions, and proximal regions. Ten high-quality spreads revealed metaphase dynamics from the beginning to the end and the transition to anaphase. Chromosomes gradually grew larger and thicker into linked chromatids, which grew more significantly in width than in length. Based on the combination of 5S rDNA and (AG_3_T_3_)_3_ signal patterns, ten chromosomes were exclusively distinguished, and the remaining twelve chromosomes were divided into two distinct groups. Our physical map, which can reproduce dynamic metaphase progression and distinguish chromosomes, will powerfully guide cytogenetic research on *Chimonanthus* and other trees.

## 1. Introduction

Fragrant species of *Chimonanthus* L. (Calycanthaceae) that are endemic to China and on The Plant List include six accepted taxa. *Chimonanthus campanulatus* R.H. Chang & C.S. Ding was established as a new species by Chang and Ding [1]. The *Chimonanthus* chromosome number (2*n* = 22) was first reported by Sugiura [2]. *Ch. campanulatus* has a 2*n* = 2*x* = 22 = 20 m (2SAT) + 2 sm karyotype [3]. The other five species, namely, *Chimonanthus grammatus* M.C. Liu, *Chimonanthus nitens* Oliv., *Chimonanthus nitens* var. *salicifolius* (S.Y. Hu) H.D. Zhang, *Chimonanthus praecox* (L.) Link, and *Chimonanthus zhejiangensis* M.C. Liu, all share a 2*n* = 2*x* = 22 = 20 m + 2 sm karyotype [3,4,5]. However, *Ch. nitens* also has a 2n = 2*x* = 22 = 18 m + 4 sm karyotype [4]. Among these six *Chimonanthus* species, *Ch. campanulatus* is the only one that possesses one pair of satellite chromosomes. However, one pair of satellite chromosomes was also observed in the same family (Calycanthaceae) but a different genus (*Calycanthus*): *Calycanthus chinensis* (W.C. Cheng & S.Y. Chang) P.T. Li, with a karyotype of 2*n* = 2*x* = 22 = 18 m + 2 m (SAT) + 2 sm [6], and *Calycanthus occidentalis* Hook. & Arn., with a karyotype of 2*n* = 22 = 20 m (2SAT) + 2 sm [7]. The genome size of *Ch. campanulatus* is unknown, but that of *Ch. praecox* is ~841 Mb [8]. In the same family (Calycanthaceae) but a different genus (*Calycanthus*), the species *Calycanthus floridus* L. shows a genome size of ~958 Mb [9] and a karyotype of 2*n* = 22 = 22 m [3]. Hence, genomic and chromosome information in *Chimonanthus* is rare and needs to be further sought.

Chen [10] studied the biosystematics of species in the genus *Chimonanthus* and inferred a close relationship between *Ch. campanulatus* and *Ch. praecox*. Dai [11] analysed the phylogeography, phylogeny and genetic diversity of species in the genus *Chimonanthus* by inter-simple sequence repeats (ISSRs), chloroplast DNA (*trn*L-F, *trn*S-G, and *trn*H-*psb*A), and the internal transcribed spacer (ITS) and speculated that current populations evolved independently in their respective refuges, e.g., *Ch. campanulatus* in Yunnan. Shu et al. [12] reviewed the non-volatile components and pharmacology of species in the genus *Chimonanthus* and found no reports on *Ch. campanulatus*. Similarly, other types of studies on *Ch. campanulatus* are also quite scarce. 

To date, most studies of *Chimonanthus* have focused on *Ch. praecox* and *Ch. nitens*, while only a few studies have reported on *Ch. nitens* var. *salicifolius*, *Ch. grammatus*, and *Ch. zhejiangensis*. Studies on *Ch. praecox* have focused on transcriptomic and proteomic profiling throughout flower development [13,14,15,16]; fragrance gene identification [17]; floral scent emission from nectaries on the adaxial side of the innermost and middle petals [18]; separation and determination of volatile compounds [19,20], phenolic compounds [21], alkaloids and flavonoids [22,23,24], and sesquiterpenoids [25]; in vitro culture system development [26]; genetic linkage map construction [27]; simple sequence repeat (SSR) [28], expressed sequence tag (EST) [29], and amplified fragment length polymorphism (AFLP) [30] development; and *ANL2* [31], *CpAGL2* [32], *CpAP3* [33], *CpCAF1* [34], *CpCZF1/2* [35], *Cpcor413pm1* [36], *CpEXP1* [37], *CpH3* [38], *CpLEA5* [39], *Cplectin* [29], *CpNAC8* [40], *CpRALF* [41], *CpRBL* [42], *FPPS* [43], and *G6PDH1* [44] cloning and development. Studies on *Ch. nitens* have focused on its calycanthine, chimonanthine, coumarins, flavonoids, phenolic acids, terpenoids, and volatile oils [45,46,47] and their pharmacologies (anti-inflammatory properties [48], antihyperglycaemic [49] and antihyperlipidaemic efficacies, and antioxidant capacity [50], inhibitory α-glucosidase activity [51], and toxicity [52]), phylogeography [45,53], and fingerprints [54,55]. Studies on *Ch. nitens* var. *salicifolius* have focused on leaf and flower transcriptome profiling [56], fingerprinting [57], sesquiterpenoids [21], nor-sesquiterpenoids [8], volatile oils and cineole [58], and the protective effect of leaves against 5-fluorouracil-induced gastrointestinal mucositis [54]. Studies on *Ch. grammatus* have focused on its β-sitosterol, quercetin, kaempferol, and isofaxidin [59] and essential oils [60]. Studies on *Ch. zhejiangensis* have focused only on its essential oils [61]. No related studies were found for *Ch. campanulatus*.

Since the number of studies on *Ch. campanulatus* is currently quite small, in this study, fluorescence in situ hybridization (FISH) was used to analyse mitotic metaphase chromosomes of *Ch. campanulatus* with the oligonucleotides 5S rDNA and (AG_3_T_3_)_3_. The aim was to construct a physical map and distinguish the chromosomes of *Ch. campanulatus*, which will aid in molecular genetic map construction and pharmacological studies in *Ch. campanulatus*.

## 2. Materials and Methods

### 2.1. Chromosomes and Probe Preparation

Seeds of *Ch. campanulatus* R.H. Chang & C.S. Ding were collected from Chengdu Botanical Garden and germinated in wet sand at room temperature (15–25 °C). When the roots reached a length of 2 cm, the root tips were excised and immediately treated with nitrous oxide for four hours. Later, the root tips were transferred to 100% acetic acid for 5 min. Next, the meristems of the root tips were treated with cellulase and pectinase (1 mL buffer + 0.04 g cellulase + 0.02 g pectinase, the buffer 50 mL was included 0.5707 g trisodium citrate + 0.4324 g citric acid), which were produced by Yakult Pharmaceutical Ind. Co., Ltd. (Tokyo, Japan) and Kyowa Chemical Products Co., Ltd. (Osaka, Japan), and then placed into suspension for dropping onto slides. Finally, air-dried slides were examined using an Olympus CX23 microscope (Olympus, Japan), and high-quality spreads were further used in a follow-up experiment.

Two oligonucleotides, namely, 5S rDNA [62] and (AG_3_T_3_)_3_ [63], were used in this study. The probes were synthesized by Sangon Biotechnology Limited Corporation (Shanghai, China), and their 5′ ends were labelled by carboxyfluorescein (FAM) or carboxytetramethylrhodamine (TAMRA). The synthetic probes were dissolved in ddH_2_O and maintained at a concentration of 10 μM.

### 2.2. Hybridization and Image Capture

Hybridization was performed as previously described by Luo et al. [62]. High-quality spreads on slides were each subjected to a series of fixation (4% paraformaldehyde), dehydration (75%, 95%, 100% ethanol), degeneration (Deionized formamide, 80 °C), and a second dehydration (−20 °C), added to 10 μL of hybridization mixture [0.325 μL of 5S rDNA, 0.325 μL of (AG_3_T_3_)_3_, 4.675 μL of 2× SSC, and 4.675 μL of ddH_2_O], and incubated at 37 °C for 2 h. Subsequently, hybridized chromosomes were washed with 2× SSC and ddH_2_O twice for 5 min at room temperature, air-dried, and counterstained with 4,6-diamidino-2-phenylindole (DAPI, Vector Laboratories, Inc., Burlingame, CA, USA).

The slides were examined by an Olympus BX63 fluorescence microscope equipped with a Photometric SenSys Olympus DP70 CCD camera (Olympus, Japan). Approximately 60 metaphases from ten slides of ten *Ch. campanulatus* root tips were observed in this study. Greater than 30 metaphases in which the chromosomes were well separated were selected to count the chromosome number. Ten better spreads were used for karyotype analysis. Single chromosomes were isolated using Photoshop 7.0 (Adobe Systems Incorporated, San Jose, CA, USA), and each spread was measured three times to provide consistent karyotype data. Chromosomes in the physical maps were aligned based on length and signal patterns. Karyotype idiograms were constructed in Excel 2019 and PowerPoint 2019 based on the relative chromosome lengths.

## 3. Results

### 3.1. S rDNA and (AG_3_T_3_)_3_ Enabled Visualization of Ch. Campanulatus Chromosomes

**Figure 1:**
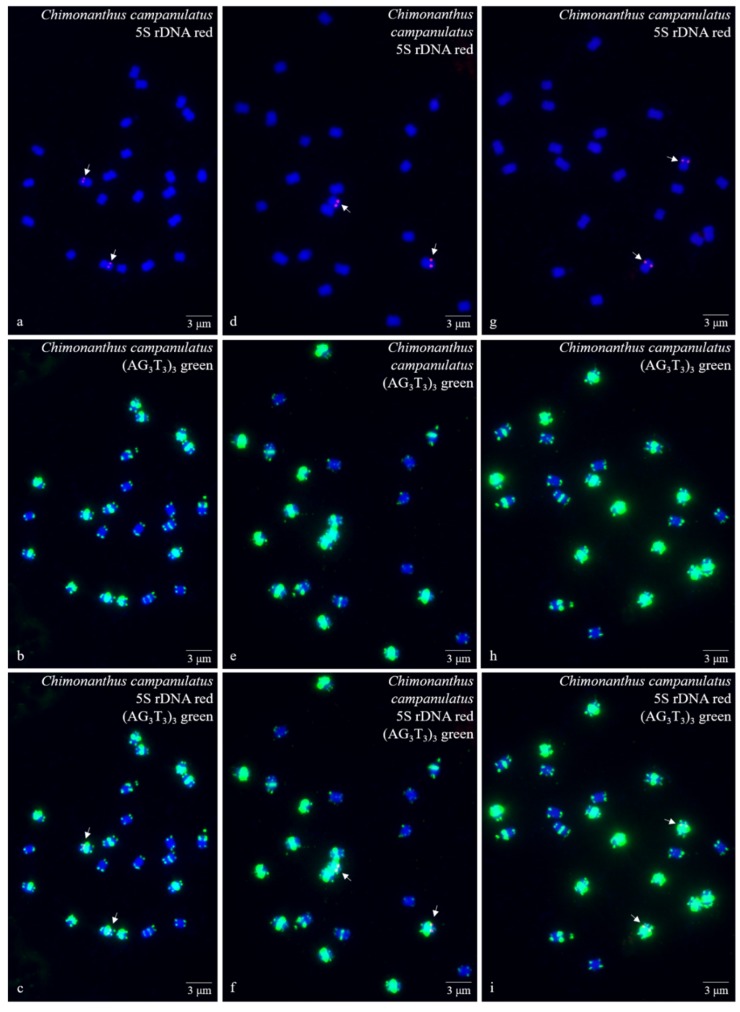
Mitotic metaphase (preliminary stage) chromosomes of *Chimonanthus campanulatus* R.H. Chang & C.S. Ding after fluorescence in situ hybridization (FISH). Three spreads are presented in Figure 1**a**–**c**, Figure 1**d**–**f**, and Figure 1**g**–**i**. The probe oligo–5S rDNA result with chromosomes visualized by carboxytetramethylrhodamine (TAMRA) (red, white arrows) is shown in Figure 1**a**,**c**,**d**,**f**,**g**,**i**, whereas the probe oligo–(AG_3_T_3_)_3_ result with chromosomes visualized by carboxyfluorescein (FAM) (green) is shown in Figure 1**b**,**c**,**e**,**f**,**h**,**i**. Chromosomes were counterstained by 4,6-diamidino-2-phenylindole (DAPI) (blue) in all images. Scale bar = 3 μM.

**Figure 2:**
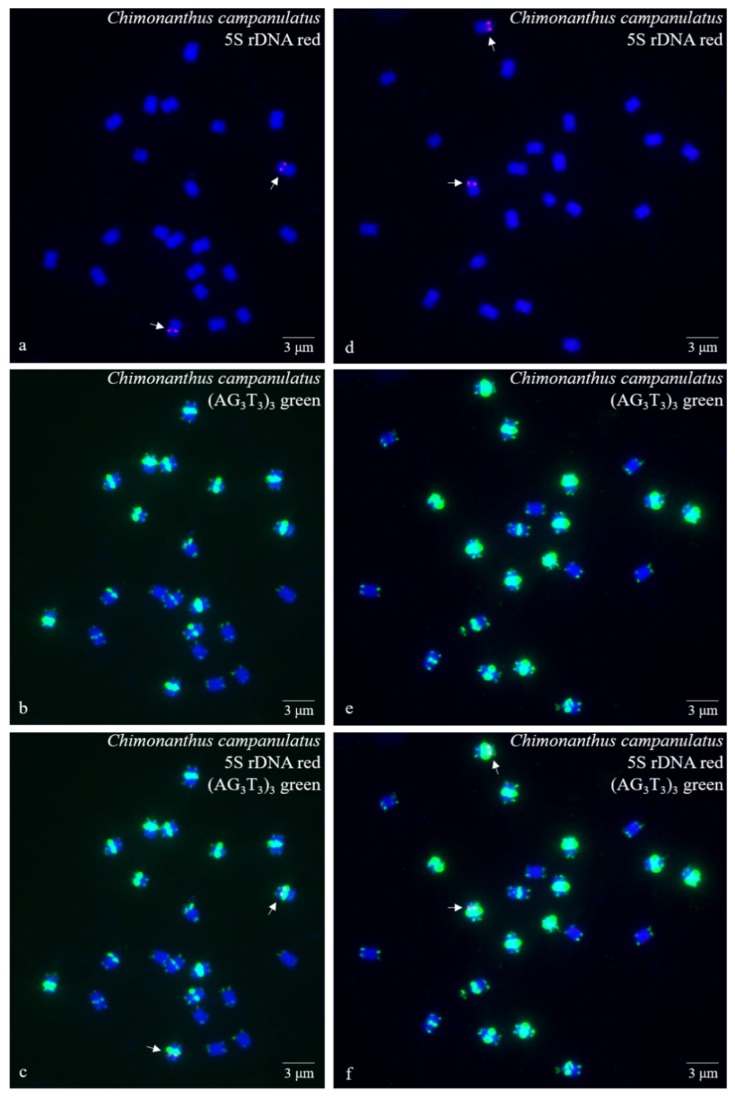
Mitotic metaphase (development stage) chromosomes of *Chimonanthus campanulatus* after FISH. Two spreads are presented in Figure 2**a**–**c** and Figure 2**d**–**f**. The probe oligo–5S rDNA result with chromosomes visualized by TAMRA (red, white arrows) is shown in Figure 2**a**,**c**,**d**,**f**, whereas the probe oligo–(AG_3_T_3_)_3_ result with chromosomes visualized by FAM (green) is shown in Figure 2**b**,**c**,**e**,**f**. Chromosomes were counterstained by DAPI (blue) in all images. Scale bar = 3 μM.

**Figure 3:**
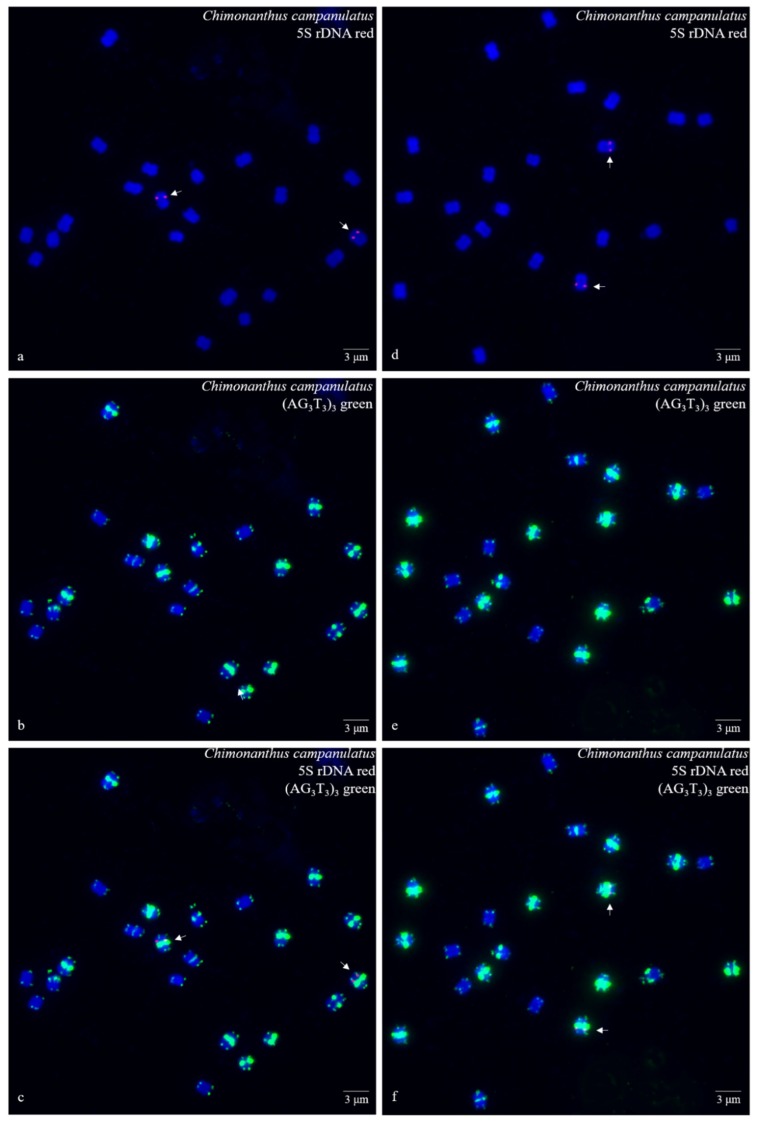
Mitotic metaphase (further development stage) chromosomes of *Chimonanthus campanulatus* after FISH. Two spreads are presented in Figure 3**a**–**c** and Figure 3**d**–**f**. The probe oligo-5S rDNA result with chromosomes visualized by TAMRA (red, white arrows) is shown in Figure 3**a**,**c**,**d**,**f**, whereas the probe oligo–(AG_3_T_3_)_3_ result with chromosomes visualized by FAM (green) is shown in Figure 3**b**,**c**,**e**,**f**. Chromosomes were counterstained by DAPI (blue) in all images. Scale bar = 3 μM.

**Figure 4:**
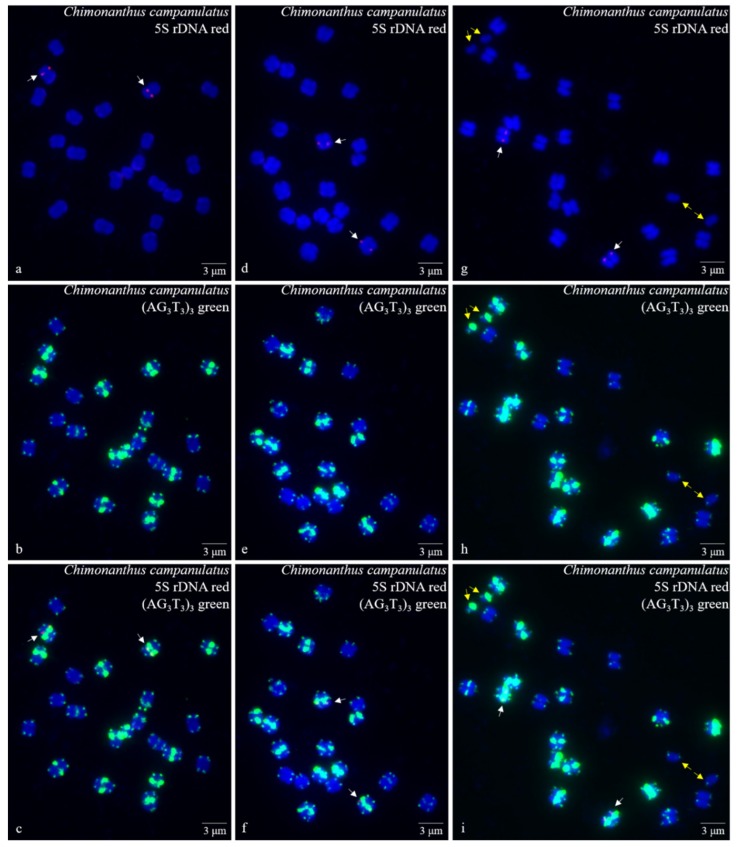
Mitotic metaphase (final stage in Figure 4**a**–**f**, end of metaphase to beginning of anaphase in Figure 4**g**–**i**) chromosomes of *Chimonanthus campanulatus* after FISH. Three spreads are presented in Figure 4**a**–**c**, Figure 4**d**–**f**, and Figure 4**g**–**i**. The probe oligo–5S rDNA result with chromosomes visualized by TAMRA (red, white arrows) is shown in Figure 4**a**,**c**,**d**,**f**,**g**,**i**, whereas the probe oligo–(AG_3_T_3_)_3_ result with chromosomes visualized by FAM (green) is shown in Figure 4**b**,**c**,**e**,**f**,**h**,**i**. Two chromosomes have split into four chromosomes in Figure 4**g**–**i** (yellow arrows), and 20 chromosomes are preparing to split, which shows that this spread was at the end of metaphase to beginning of anaphase. Chromosomes were counterstained by DAPI (blue) in all images. Scale bar = 3 μM.

**Figure 5:**
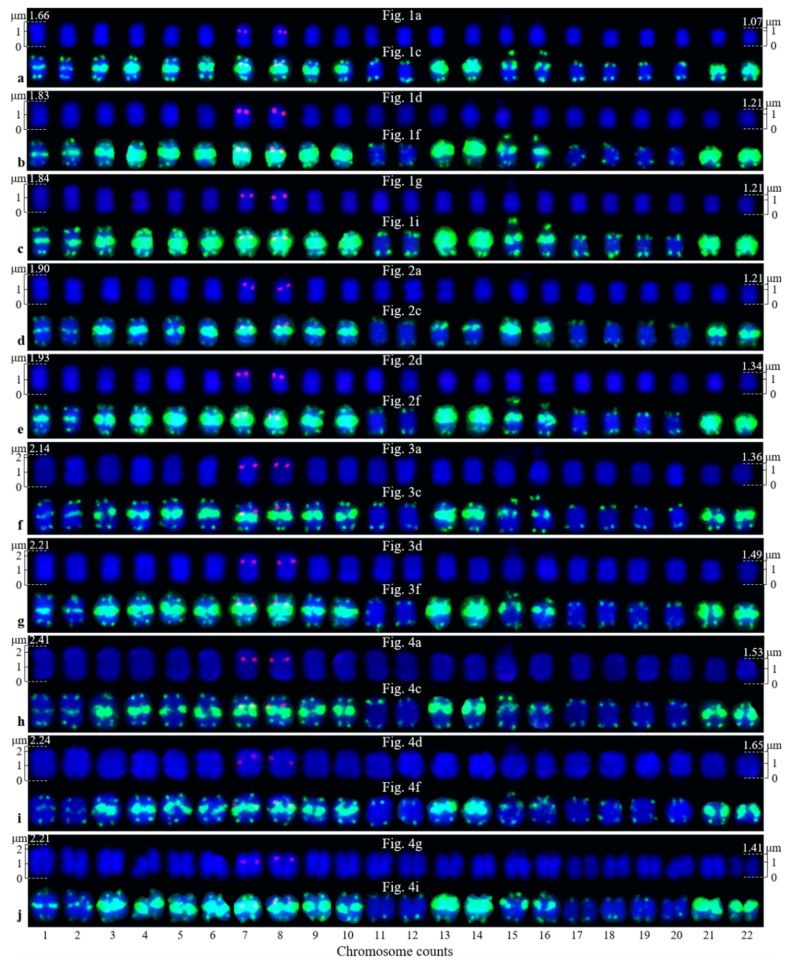
Each chromosome of *Chimonanthus campanulatus* isolated from Figure 1, Figure 2, Figure 3 and Figure 4. For example, the chromosomes in Figure 5**a** were isolated from Figure 1a and Figure 1c, as indicated in the middle of each chromosome line. Because the (AG_3_T_3_)_3_ end signals likely affect the measurement of chromosome length, only the first and last chromosomes in Figure 1a,d,g, Figure 2a,d, Figure 3a,d, Figure 4a,d,g were measured for total length. The chromosomes were aligned based on length. The bottom numbers indicate chromosome counts. The four split chromosomes in Figure 4g,i (yellow arrows) were assembled into two chromosomes (17 and 22) based on their signal patterns.

Ten high-quality spreads of mitotic metaphase chromosomes of *Ch. campanulatus* after FISH are illustrated in Figure 1 (preliminary stage), Figure 2 (development stage), Figure 3 (further development stage), and Figure 4 (final stage in Figure 4a–f, end of metaphase to beginning of anaphase in Figure 4g–i), which exhibited dynamic metaphase progression from the preliminary stage to the final metaphase and confirmed the repeatability and stability of our FISH results. In total, twenty-two chromosomes were counted in each spread except the last spread, as shown in Figure 4g–i (twenty-four chromosomes). In the last spread, chromosomes were at the end of metaphase to the beginning of anaphase, with twenty chromosomes were preparing to split (chromatids) and two chromosomes had already split into four chromosomes (yellow arrows in Figure 4g–i). To better describe the chromosome characteristics, a single chromosome was isolated from Figure 1, Figure 2, Figure 3 and Figure 4 and illustrated in Figure 5. Chromosomes of each spread were aligned by their length from longest (chromosome 1) to shortest (chromosome 22) and their signal patterns. The four split chromosomes were assembled into two chromosomes (17 and 22) based on their signal patterns (Figure 5). Hence, all ten spreads showed 22 chromosomes.

Figure 5 also shows the lengths of the longest (1) and shortest chromosomes (22) in the ten *Ch. campanulatus* spreads originally shown in Figure 1a,d,g, Figure 2a,d, Figure 3a,d, and Figure 4a,d,g, with the lengths ranging from 1.66–1.07 μM, 1.83–1.21 μM, 1.84–1.21 μM, 1.90–1.21 μM, 1.93–1.34 μM, 2.14–1.36 μM, 2.21–1.49 μM, 2.41–1.53 μM, 2.24–1.65 μM, and 2.21–1.41 μM, respectively. There was a very significant difference in chromosome length among these ten spreads (*p* = 0.00019), revealing dynamic chromosome growth in terms of length from the preliminary stage to the end of metaphase. The longest chromosome was 2.41 μM long, i.e., still less than 3 μM; hence, all of the chromosomes were small chromosomes. Due to the small size and unclear centromere locations of the chromosomes, it was difficult to determine the long arms and short arms, and karyotype analysis was not performed further.

Figure 5 further displays a conserved 5S rDNA signal distribution and a diverse (AG_3_T_3_)_3_ signal distribution for *Ch. campanulatus*. Weak 5S rDNA signals were observed only in proximal regions of two chromosomes (7/8) (red colour in Figure 1a,c,d,f,g,i, Figure 2a,c,d,f, Figure 3a,c,d,f, Figure 4a,c,d,f,g,i, and Figure 5), which were adjacent to the (AG_3_T_3_)_3_ proximal signals on these two chromosomes. Weak (AG_3_T_3_)_3_ signals were observed on both chromosome ends (green colour in Figure 1b,c,e,f,h,i, Figure 2b,c,e,f, Figure 3b,c,e,f, Figure 4b,c,e,f,h,i, and Figure 5), which ensured accurate chromosome counts. Weak (AG_3_T_3_)_3_ signals were also observed in the proximal regions of two chromosomes (1/2), and strong (AG_3_T_3_)_3_ signals were observed in the proximal regions of two chromosomes (15/16). Interestingly, a pair of satellite bodies was observed on chromosomes 15/16, as shown by the upper-chromosome end (AG_3_T_3_)_3_ signals located far away from the established ends of the two chromosomes (15/16). Meanwhile, very strong (AG_3_T_3_)_3_ signals were observed in the proximal regions of eight chromosomes (3/4/5/6/7/8/9/10) and in the subtelomeric regions of four chromosomes (13/14/21/22). Finally, almost no (AG_3_T_3_)_3_ signals were observed in the proximal regions of six chromosomes (11/12/17/18/19/20).

### 3.2. Physical Map Reproduced Metaphase Dynamics and Distinguished Chromosomes

**Figure 6:**
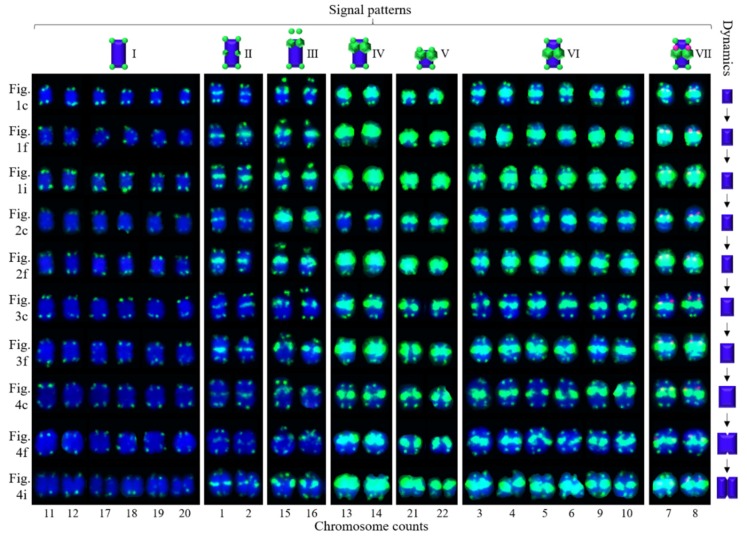
Physical map of *Chimonanthus campanulatus*. Each chromosome was isolated from Figure 5. The left indicates the origins of each line of chromosomes. The right metaphase dynamic ideograms were constructed based on the metaphase dynamics of chromosomes shown on the left. The signal pattern ideograms at the top were constructed based on the signal patterns of chromosomes. The numbers at the bottom indicate chromosome counts. Chromosomes 15 and 16 are SAT chromosomes.

To better describe the chromosomes of *Ch. campanulatus*, the chromosome hybridizations with 5S rDNA and (AG_3_T_3_)_3_ shown in Figure 5 were separated to create Figure 6. The metaphase dynamic idiograms on the right were constructed based on the metaphase dynamics of chromosomes on the left. Although this dynamic metaphase progression is not a novel mitotic phenomenon, it still provides the first visualization of the dynamic metaphase progression of *Ch. campanulatus*, from the preliminary stage to the end of metaphase and from single-chromosome duplication to the linkage or splitting of chromosomes. In addition, obvious satellite bodies gradually moved from far away from the arm ends to close to the arm ends from the beginning to the end of metaphase.

Furthermore, idiograms of the seven types of 5S rDNA and (AG_3_T_3_)_3_ signal patterns observed for *Ch. campanulatus* chromosomes (top of Figure 6) were constructed: Type I: Weak (AG_3_T_3_)_3_ signals on both chromosome ends were observed for six chromosomes (11/12/17/18/19/20). Type II: Weak (AG_3_T_3_)_3_ signals on both chromosome ends and in the proximal regions were observed for two chromosomes (1/2). Type III: Weak (AG_3_T_3_)_3_ signals on both chromosome ends and strong (AG_3_T_3_)_3_ signals in the distal regions were observed for two chromosomes (15/16). A peculiar phenomenon was observed for these two chromosomes: Satellite bodies were shown by upper-end signals located far away from the designated chromosome ends. Type IV: Weak (AG_3_T_3_)_3_ signals on both chromosome ends and very strong (AG_3_T_3_)_3_ signals in the subtelomeric regions (adjacent to the end signals) were observed for two chromosomes (13/14). Type V: Weak (AG_3_T_3_)_3_ signals on both chromosome ends and very strong (AG_3_T_3_)_3_ signals in the subtelomeric regions (adjacent to the end signals) were observed for the two shortest chromosomes (21/22). Type VI: Weak (AG_3_T_3_)_3_ signals on both chromosome ends and very strong (AG_3_T_3_)_3_ signals in the proximal regions were observed for six chromosomes (3/4/5/6/9/10). Type VII: Weak (AG_3_T_3_)_3_ signals on both chromosome ends and very strong (AG_3_T_3_)_3_ signals in the proximal regions, as well as 5S rDNA signals adjacent to proximal (AG_3_T_3_)_3_ signals, were observed for two chromosomes (7/8). In contrast to types I and VI, which included six chromosomes, types II, III, IV, V, and VII each included only two chromosomes. Therefore, 5S rDNA and (AG_3_T_3_)_3_ signal patterns exclusively distinguished ten chromosomes, namely, 1/2/7/8/13/14/15/16/21/22, and divided the other twelve chromosomes into two obvious groups, namely, 3/4/5/6/9/10 and 11/12/17/18/19/20. The physical map of *Ch. campanulatus* convincingly demonstrated that (AG_3_T_3_)_3_ not only labelled chromosome ends (including satellite bodies), which are used to count chromosome numbers, but also labelled chromosome proximal regions and subtelomeric regions with obviously different signal intensities, which are used as effective FISH markers for cytogenetic analysis.

**Figure 7:**
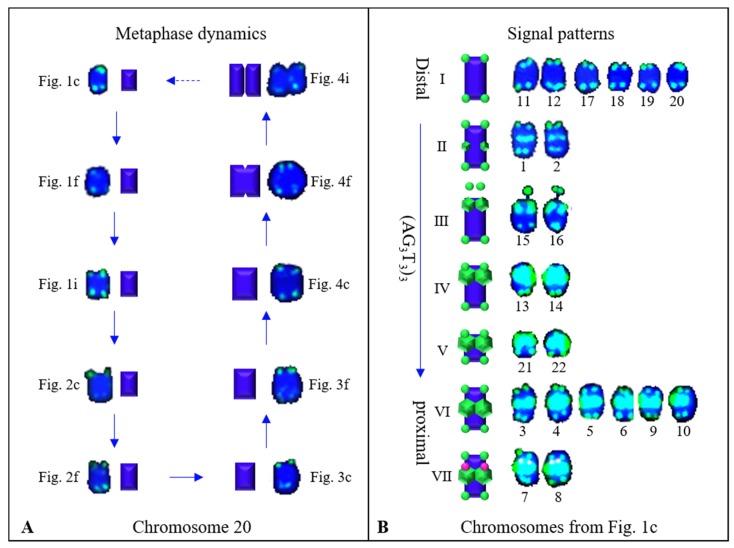
Dynamic metaphase progression and signal patterns of *Chimonanthus campanulatus* chromosomes isolated from Figure 6. The left panel (Figure 7A) shows chromosome 20 in ten spreads to present metaphase dynamics (Figure 1c,f,i, Figure 2c,f, Figure 3c,f, Figure 4c,f,i). The right panel (Figure 7B) uses the 22 chromosomes in Figure 1c to demonstrate signal patterns. The signal intensity of the distal repeat probe (AG_3_T_3_)_3_ was variable, ranging from weak on the chromosome ends to strong in the proximal regions.

In summary, Figure 7 shows the refined metaphase dynamics and signal patterns of *Ch. campanulatus* chromosomes isolated from Figure 6. Chromosome 20, represented by ten spreads (visualized in Figure 1c,f,i, Figure 2c,f, Figure 3c,f, Figure 4c,f,i), presented dynamic metaphase progression from the beginning to the end. Chromosomes gradually grew larger and wider to form linked chromatids, which grew more significantly in width than in length (Figure 7A). The chromosomes shown in Figure 1c exhibited seven types of signal patterns. The intensity of (AG_3_T_3_)_3_ signals showed quite high diversity, ranging from weak to very strong as follows: far away from the ends (satellite bodies), ends, subtelomeric regions, and proximal regions (Figure 7B). Based on a combination of 5S rDNA and (AG_3_T_3_)_3_ signal patterns, ten chromosomes were exclusively distinguished, and the remaining twelve chromosomes were divided into two obvious groups. In the future, we will explore more oligonucleotides to discern the twelve chromosomes categorized as types I and VII.

## 4. Discussion

### 4.1. Karyotype Analysis and Dynamic Metaphase Progression

Ten high-quality spreads displayed a very significant difference in chromosome length, which revealed chromosome dynamic growth in terms of length from the preliminary stage to the end of metaphase and transition to anaphase. The corresponding physical map of chromosomes reproduced the dynamic mitotic metaphase progression in *Ch*. *campanulatus* for the first time. Similarly, Matsui [64] examined dynamic changes in proteins during mitotic metaphase, anaphase, and telophase in human epithelial HeLa S3 cell populations to reproduce dynamic mitotic progression. The length of *Ch*. *campanulatus* chromosomes was 1.07–2.41 μM in this study. In previous works, chromosome lengths were reported to be 1.05–1.81 μM in *Ligustrum lucidum* Lindl. [65], 1.12–2.06 μM in *Fraxinus pennsylvanica* Marsh. [65], 1.22–2.11 μM in *Fragaria nilgerrensis* Schlecht. ex Gay [66], 1.23–2.34 μM in *Zanthoxylum armatum* Candelle [67], 1.25–2.83 μM in *Ligustrum* × *vicaryi* Rehder [65], 1.48–2.08 μM in *Fragaria vesca* L. [66], 1.50–2.32 μM in *Syringa oblata* Ait. [65], 1–4 μM in *Rubus* L. species [68], 1.82–2.75 μM in *Berberis diaphana* Maxim. [69], 4.03–7.21 μM in *Piptanthus concolor* Harrow ex Craib [62], 4–13 μM in *Avena sativa* L. [70], and 9–13 μM in *Triticum aestivum* L. ‘Chinese Spring’ [71]. In our unpublished works, chromosome lengths were recorded to be 0.97–2.16 μM in *Juglans regia* L., 0.98–2.65 μM in *Juglans sigillata* Dode, 1.13–2.41 μM in *Firmiana platanifolia* (L. f.) Marsili, 1.12–2.49 μM in *Koelreuteria bipinnata* Franch., 1.31–1.43 μM in *Robinia pseudoacacia* L., 1.44–5.28 μM in *Podocarpus macrophyllus* (Thunb.) D. Don, 1.75–2.23 μM in *Erythrina crista-galli* L., 2.05-3.70 μM in *Croton tiglium* L., 2.16–4.96 μM in *Quercus aquifolioides* Rehd. et Wils., and 8.73–14.35 μM in *Cycas revoluta* Thunb. The length of the longest chromosome in *Ch*. *campanulatus* was approximately equal to that in *Z. armatum* (2.34 μM), while the length of the shortest chromosome in *Ch*. *campanulatus* was approximately equal to that in *L. lucidum* (1.05 μM).

In this study, twenty-two chromosomes were observed in *Ch*. *campanulatus*, which was in agreement with the numbers reported in previous works [2,3]. Similar to the results from Liu et al. [3], one pair of satellite chromosomes was clearly observed in this study. Satellite bodies, as hereditary features, may be used to distinguish species [72,73]. The other five species in the genus *Chimonanthus* (*Ch. grammatus*, *Ch. nitens*, *Ch. nitens* var. *salicifolius*, *Ch. praecox*, and *Ch. zhejiangensis*) do not possess satellite bodies, based on the findings of previous works [3,4,5], revealing relatively distant relationships between *Ch. campanulatus* and the other five *Chimonanthus* species. However, in the same family (Calycanthaceae) but a different genus (*Calycanthus*), the species *Ca. chinensis* and *Ca. occidentalis* possess one pair of satellite chromosomes and twenty-two chromosomes [6,7], indicating moderately close relationships between these two species and *Ch. campanulatus*.

### 4.2. Distinguishing Ch. campanulatus chromosomes

No FISH technology has been previously applied in *Chimonanthus* species. Here, (AG_3_T_3_)_3_ and 5S rDNA were tested in *Ch*. *campanulatus* for the first time. In contrast to 5S rDNA, (AG_3_T_3_)_3_ generally labels only chromosome ends, rendering it a less effective FISH marker for distinguishing chromosomes [65,69,74]. However, in this study, (AG_3_T_3_)_3_ was an excellent marker because it distinguished *Ch*. *campanulatus* chromosomes based on its location: far away from chromosome ends (satellite bodies), ends, subtelomeric regions and proximal regions; in this order, its intensity ranged from weak to strong and very strong. Such high diversity in (AG_3_T_3_)_3_ signal intensity has rarely been found in other species, but this oligonucleotide was found to locate to the proximal region of chromosomes in *Zea mays* L. [63], *Podocarpus* L. Her. ex Persoon species [75], and *Philodendron* Schott species [76].

In this study, (AG_3_T_3_)_3_ labelling of satellite bodies in *Ch*. *campanulatus* was reported for the first time. Satellite bodies of *B. diaphana* are a quarter of the chromosome size (~0.6 μM) and are labelled at the ends by (AG_3_T_3_)_3_ [69]. Satellite bodies are often connected to the main body of the chromosome by very lightly staining strands [72]. These bodies vary in size according to the position of the secondary constriction. If the secondary constriction is very close to an end of the chromosome, the satellite may be a barely perceptible dot [73]. In this study, the satellite bodies were small, barely perceptible dots (~0.1 μM) located far away from their designated ends and were obvious based on their green (AG_3_T_3_)_3_ signals. Hence, (AG_3_T_3_)_3_ may aid in the visualization of such unobservable satellite bodies.

The 5S rDNA in this study was quite conserved, located only adjacent to (AG_3_T_3_)_3_ proximal regions on two chromosomes. Similarly, 5S rDNA distinguished two chromosomes in *B. diaphana* [69] and *C. tiglium*, *C. revoluta*, *E. crista-galli*, *J. regia*, *J. sigillata*, *K. bipinnata*, *Q. aquifolioides* and *P. macrophyllus* (unpublished data); four chromosomes in *Berberis soulieana* [69], *F. pennsylvanica* [65], *Z. armatum* [67], and *L. baviensis* (unpublished data); six chromosomes in *L. lucidum* and *L.* × *vicaryi* [65] and *L. elongata* and *R. pseudoacacia* (unpublished data); and eight chromosomes in *S. oblata* [65] and *F. platanifolia* (unpublished data). In contrast, 5S rDNA displayed quite high diversity and distinguished sixteen chromosomes in *P. concolor* [62]. Therefore, (AG_3_T_3_)_3_ and 5S rDNA both have the potential to effectively discern chromosomes as FISH markers.

## 5. Conclusions

In this study, based on the combination of (AG_3_T_3_)_3_ and 5S rDNA signal patterns, only ten chromosomes were exclusively discerned among the twenty-two chromosomes of *Ch*. *campanulatus.* The remaining twelve chromosomes were divided into two obvious groups. It is necessary to explore more oligonucleotide probes to further distinguish *Ch*. *campanulatus* chromosomes and establish detailed physical maps.
